# Insights on the Organ-Dependent, Molecular Sexual Dimorphism in the Zebra Mussel, *Dreissena polymorpha*, Revealed by Ultra-High-Performance Liquid Chromatography–Tandem Mass Spectrometry Metabolomics

**DOI:** 10.3390/metabo13101046

**Published:** 2023-10-01

**Authors:** Emilie Lance, Lucas Sartor, Pierre Foucault, Alain Geffard, Benjamin Marie

**Affiliations:** 1 UMR MNHN/CNRS Molécules de Communication et Adaptations des Microorganismes (MCAM), Muséum National d’Histoire Naturelle, 75005 Paris, Francepierre.foucault@mnhn.fr (P.F.); benjamin.marie@mnhn.fr (B.M.); 2 UMR-I 02 SEBIO, University of Reims, BP 1039, CEDEX 2, 51687 Reims, France; alain.geffard@univ-reims.fr

**Keywords:** metabolome, zebra mussel, gender and organ specificity, ecotoxicology

## Abstract

The zebra mussel, *Dreissena polymorpha*, is extensively used as a sentinel species for biosurveys of environmental contaminants in freshwater ecosystems and for ecotoxicological studies. However, its metabolome remains poorly understood, particularly in light of the potential molecular sexual dimorphism between its different tissues. From an ecotoxicological point of view, inter-sex and inter-organ differences in the metabolome suggest variability in responsiveness, which can influence the analysis and interpretation of data, particularly in the case where males and females would be analyzed indifferently. This study aimed to assess the extent to which the molecular fingerprints of functionally diverse tissues like the digestive glands, gonads, gills, and mantle of *D. polymorpha* can reveal tissue-specific molecular sexual dimorphism. We employed a non-targeted metabolomic approach using liquid chromatography high-resolution mass spectrometry and revealed a significant sexual molecular dimorphism in the gonads, and to a lesser extent in the digestive glands, of *D. polymorpha*. Our results highlight the critical need to consider inter-sex differences in the metabolome of *D. polymorpha* to avoid confounding factors, particularly when investigating environmental effects on molecular regulation in the gonads, and to a lesser extent in the digestive glands.

## 1. Introduction

Ecotoxicological and biosurvey studies often propose the use of biological markers or specific endpoints measured in model organisms as an indication of exposure to natural or anthropogenic substances. Thus, one great challenge of ecotoxicology and stress ecology research is to find relevant molecular signatures of specific stressors in sentinel organisms and to understand their physiological effects on biota. Metabolomics is a sensitive, currently emerging, high-throughput approach for investigating metabolites of low molecular weight (<1500 Da), often using nuclear magnetic resonance (NMR) or liquid/gas chromatography combined with tandem mass spectrometry (LC-MS/MS or GC–MS/MS). Metabolomics analysis allows researchers to describe the metabolite profile of an organism and determine its involvement in dynamic cellular processes to obtain an instantaneous fingerprint of the physiological state of the organism. Metabolomics applied to an organ or an entire organism constitutes one of the most reliable methods of chemical phenotyping for investigating the homeostatic responses to environmental stresses from multiple origins [1,2,3] in organisms like marine or freshwater bivalves [4,5,6,7]. Metabolomics greatly helps in characterizing the molecular effects of different stressors on metabolic pathways and increasing our understanding of the mechanism of impairment. Metabolomic investigations are independent of the background genomic dataset of the model organism and therefore provide data applicable to all organisms.

The freshwater zebra mussel, *D. polymorpha*, is a filter-feeding dreissenid mussel native to the Ponto-Caspian regions. This sessile bivalve lives mostly on hard substrates and is present in a wide range of habitats, from freshwater lakes and rivers to brackish estuaries [8]. *D. polymorpha* has been considered a widespread and invasive species [9,10,11,12]. It has colonized Western Europe and North America, developing large populations because of its high growth rate, and endangering freshwater biota and ecosystems. However, the ecological pressure of *D. polymorpha* has tended to decrease during the last few years concomitant with smaller populations and individual body sizes, allowing the benthic populations of native species to regain their competitiveness and return to pre-invasion densities [13]. Conversely, some ecological characteristics of this species such as its water purification capabilities and influence on rates of nutrient cycling are beneficial in mitigating the harmful effects of eutrophication [14].

*D. polymorpha* is also extensively used in laboratory experiments and as a bioindicator species for water pollution because of its great abundance, large repartition area, limited mobility, continuous filtering activity, high bioaccumulation potential and ease of handling [15,16,17,18,19,20,21]. Its wide food size range combined with filtering capacities ranging from 5 to 400 mL/mussel per hour allows *D. polymorpha* to be exposed to various anthropogenic or natural pollutants through direct gill absorption, ingestion of contaminated food or of particles on which pollutants may be adsorbed [22,23,24]. Interest in the zebra mussel as a bioindicator of environmental pollutants, pathogens or natural toxins such as metals, microplastics, organochlorine contaminants, cyanotoxins and parasites, and as a model organism in ecotoxicology studies, has been widely demonstrated [21,25,26,27,28,29,30,31,32,33,34,35]. *D. polymorpha* has been suggested as the freshwater counterpart of the marine mussel *Mytilus* sp. in biomonitoring and ecotoxicological studies [24]. It has been used as a sentinel organism of water quality in the Great Lakes since the mid-1970s in the Mussel Watch program for water quality monitoring [36,37]. Its remarkable bioaccumulation capacities, legal ecological status allowing *in situ* collection without limitation, and ease of transplantation in cages have stimulated great interest in using this organism in water quality management plans [38,39].

An NMR investigation of *D. polymorpha* [40] has underlined the usefulness of the metabolomic approach in comparison to the measurement of core biomarkers for identifying metabolites of interest in ecotoxicological investigations [38]. However, to enhance the ecotoxicological relevance of the molecular biomarkers, the reference metabolic conditions that differ among organs, developmental stages, and between the sexes must be clarified [41]. In marine mussels, various gender- and tissue-specific metabolome differences have already been reported, reflecting the specificities of the physiological responses to different stressors depending on which sex and organ are considered [7,42,43,44,45,46,47]. Therefore, attempting to define the corresponding baseline in terms of inter-sex and inter-organ metabolite concentrations may help researchers understand the individual variability observed in some ecotoxicological studies [48]. The aim of this study was to assess the specific metabolite fingerprints of various tissues (digestive gland, gonad, gills, mantle) in both males and females of *D. polymorpha* through a non-targeted metabolomic approach using ultra-high performance liquid chromatography–electrospray ionization–tandem mass spectrometry (UHPLC-ESI-MS/MS).

## 2. Materials and Methods

### 2.1. Biological Model of D. polymorpha 

To ensure an accurate comparison of the metabolomes of different tissues and genders of organisms, it is necessary to use individuals as similar as possible from the same population and of similar age and size. Thus, organisms were obtained from a single reference site (Lac-du-Der-Chantecoq 48°36′07.7″ N; 4°44′37.0″ E) and were selected according to metamorphosis on a hard substrate, a process that yielded a group of similar ages. For this study, organisms about 18 months of age (January 2021) were selected at a size of 25 ± 2 mm. Groups of 40 individuals were placed in aerated 3 L tanks (six aquaria) containing a 1:1 ratio of water from the sampling site and Cristalline^®^ spring water (Saint Yorre, France). After collection from the field, they were gradually acclimated to lab conditions of 16 ± 2 °C with a 12 h:12 h light:dark cycle for up to seven weeks. During the acclimation, the mussels were fed daily with 2.5 µL per individual of a dietary concentrate containing the microalgae Nannochloropsis salina (Nanno 3600^®^ Planktovie, Marseille, France). After the acclimation, individuals were randomly sampled from the six aquaria and sexed by gametes withdrawn from the gonads using a 1 mL syringe to obtain 12 males and 12 females. The mussels were anaesthetized for dissecting, and the digestive glands, gonads, mantle and gills were removed, weighed, individually snap frozen in liquid nitrogen and stored at −80 °C.

### 2.2. Extract Preparation from Mussel Tissues and Metabolome Analysis by Mass Spectrometry

LC-MS grade acetonitrile, methanol, and formic acid were obtained from Carlo Erba (Val-de-Rueil, France). A standard solution of Na formate (purchased from Sigma-Alrich, Saint-Quentin-Fallavier, France) was freshly prepared with Ultra-pure MilliQ^®^ water (Guyancourt, France). Analytes were extracted from the chosen tissues of *D. polymorpha* with weights varying from 22 to 130 mg depending on the type of tissue. The amount of solvent was adjusted in order to keep a ratio of 1 mL of 75%:25% UHPLC methanol: water per 100 mg of fresh tissue. Tissues were mechanically ground (GLH850 OMNI) and sonicated with an ultrasonic probe (Sonics VibraCell, 130 W, 20 kHz, 60% amplitude) for 30 s to release intracellular metabolite contents. Samples were then centrifuged at 15,300× *g* for 10 min at 4 °C. Supernatants (2 μL) were analyzed by UHPLC (Elute, Bruker or Ultimate 3000, Thermo, Waltham, MA, USA) on a PolarAdvance-II C18 column (2.5 µm pore-size) (Thermo, Waltham, MA, USA) at a 300 µL·min^−1^ flow rate with a linear gradient of acetonitrile in 0.1% formic acid (5–90% in 16 min). Analytes were subsequently ionized and analyzed using an electrospray ionization hybrid quadrupole time-of-flight high-resolution mass spectrometer (ESI-Qq-TOF Compact, Bruker, Bremen Germany, France) at a speed of 2 Hz over a range of 50–1500 *m*/*z* on positive simple MS mode and then on broad-band collision ion dissociation or positive autoMS/MS mode at a speed of 2–8 Hz over the 50–1500 *m*/*z* range with information-dependent acquisition.

The list of peaks (MS/MS spectra within 1 and 15 min of the LC gradient) was generated from recalibrated MS spectra (<0.5 ppm, with an internal calibrant of Na formate) with filtering of 5000 counts of minimal intensity and a minimal occurrence in at least 10% of all samples. All classical adducts ([M + H]+, [M + 2H]+, [M + 3H]+, [M + Na]+, [M + K]+, and [M + NH4]+) and related isotopic forms were searched and grouped together using a threshold value of 0.8 for the co-elution coefficient factor with MetaboScape 4.0 software (Bruker, Bremen, Germany). All data were acquired from the same single LC-MS run. Data QC and Blank samples (injected every 6 injections) were examined in order to ensure the reproducibility and robustness of the whole data series. Data quality in terms of intensity, retention time and mass drift of ions were carefully inspected and recalibration was automatically performed individually by the software on raw data of all samples using internal standards (reference Na formate solution) injected at the beginning of every sample acquisition. Different states of charge and adducts were grouped together and the area under the peak was determined to generate a unique global data matrix containing semi-quantification results for each analyte in all analyzed sample peaks for each analyte (characterized by the respective mean mass of its neutral form and its corresponding retention time).

The annotation of analytes was attempted from MS and MS/MS using the MetaboScape 4.0 (Bruker, Germany) with NPAtlas V1.0 structural databases [49] and the MetGem 1.3.6 [50] interrogating spectral database available on GNPS and MS-DIAL (HMDB, Massbank, EMBL, GNPS, CASMI, NIH).

### 2.3. Data Treatment, Statistical Analyses, and Annotation by Molecular Network

The two data tables with quantification of area under the peak for the different analytes (one with all analytes and one with only the annotated analytes) were evaluated using MetaboAnalyst 5 (www.metaboanalyst.ca (accessed on 5 June 2021)) for Pareto’s normalization, univariate group variance analyses (ANOVA and *t*-test with Benjamini FDR calculation), multivariate statistical methods (unsupervised principal component analysis, PCA) and data representation by heatmap with hierarchical clustering based on Euclidean distances (https://www.metaboanalyst.ca (accessed on 5 June 2021)). Quantifications of annotated analytes and of all analytes were also compared by gender and between tissues using PERMANOVA analyses applied on PCA in the vegan R package (vegan: community ecology package; https://cran.r-project.org/web/packages/vegan/index.html (accessed on 20 July 2021)). Pairwise comparisons were carried out with the pairwise Adonis R package. The significance threshold was set at *p* < 0.05. Analyte annotation was attempted using combined ion annotation with the MetaboScape software based on mass and isotopic pattern accuracy and with MetGem molecular networking based on the presence of ions in certain molecular clusters for which substantial annotation could be retrieved according to the MS/MS fragment occurrence. Previously uncharacterized analytes belonging to annotated metabolite clusters were considered potential analogue molecules.

## 3. Results and Discussion

### 3.1. Analysis of the Global Metabolome of Tissues from D. polymorpha Males and Females

**Figure 1:**
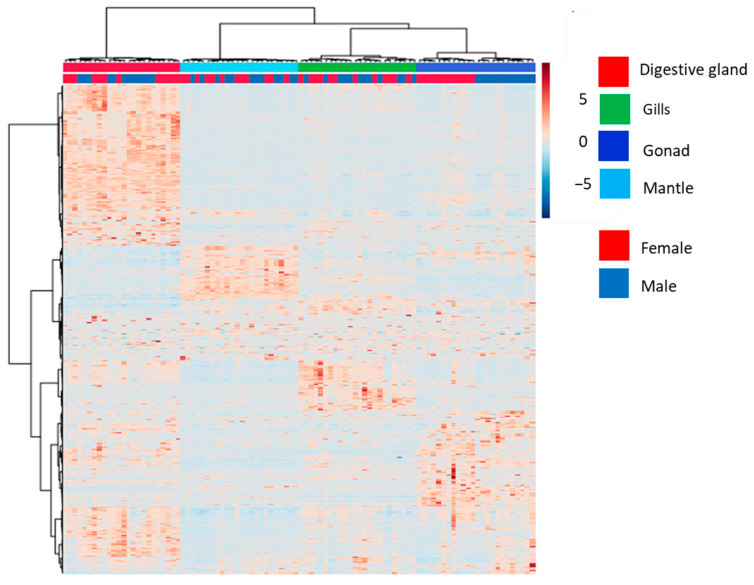
Heatmap with hierarchical classification of the whole set of metabolites (n = 2634) determined by mass spectrometry in the digestive glands (red), gills (green), gonads (dark blue), and mantle (light blue) of male (n = 12) and female (n = 12) *D. polymorpha*.

A total of 2634 analytes, annotated and not annotated, from untargeted LC-MS metabolomics of the digestive gland, gills, mantle and gonads showed that the metabolome of each tissue appeared to significantly differ from that of other tissues (PERMANOVA *p* < 0.01, Table 1A). The heatmap with hierarchical clustering revealed first that the metabolic signatures could be discriminated between the four tissues and second between males and females, with only limited intra-group individual variability (n = 12 replicates per tissue and per sex, Figure 1). The molecular profile of the gonad and of the digestive gland significantly discriminated between males and females. The sexual dimorphism of the gonad metabolome was more distinct than that of the digestive gland (PERMANOVA *p* < 0.01; PAIRWISE PERMANOVA *p* < 0.01 and *p* < 0.05, respectively, Table 1B). In the literature, clear differences in metabolomes between tissues and between the sexes have also been observed in the marine mussel *Mytilus galloprovincialis* [7,47].

**Figure 2:**
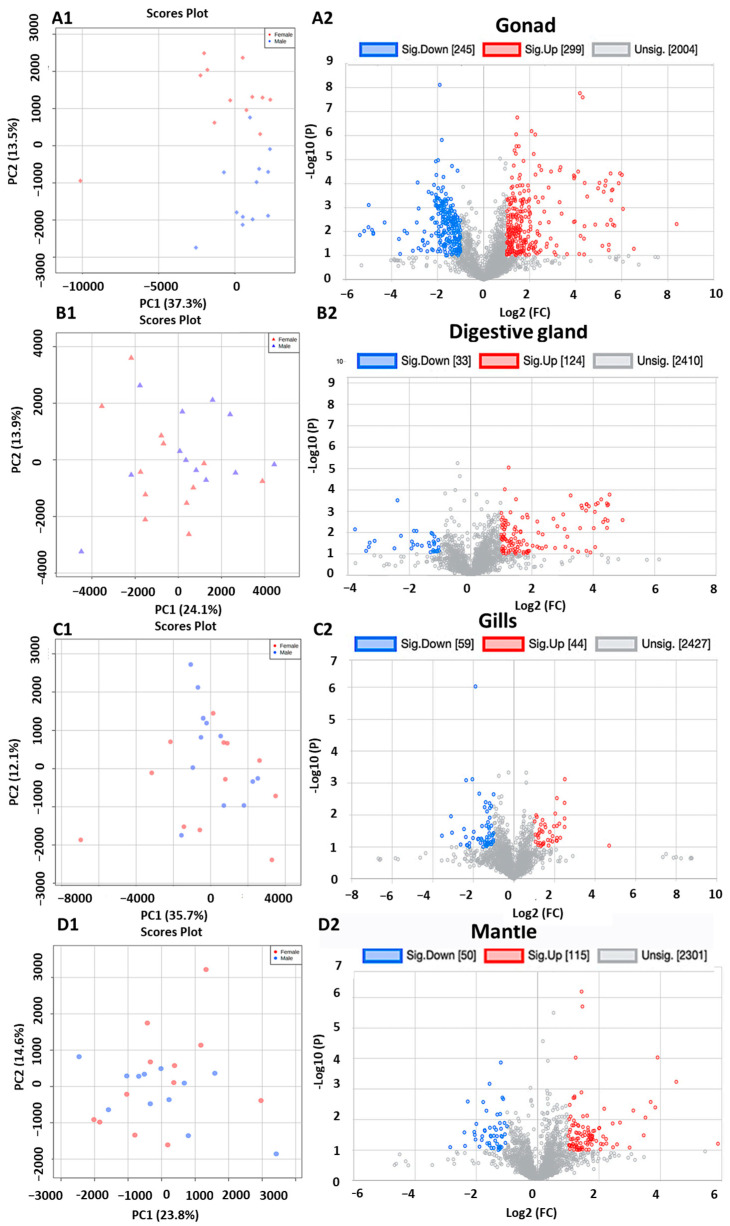
Principal component analysis (**A1**,**B1**,**C1**,**D1**) and volcano plots (**A2**,**B2**,**C2**,**D2**) of the metabolite data from gonads, digestive glands, gills, and mantles of male (n = 12, blue) and female (n = 12, red) *D. polymorpha*. The variance of the PCA is given on the axis of PC-1 and -2. In the volcano plots, the fold changes in intensity of individual metabolites between males and females are plotted on the *x*-axis (fold change ≥ 2) with the significance of the differences between males and females (*p* < 0.1) being determined by *t*-test (*y*-axis). The statistical significance for down-regulated metabolites is indicated in blue and for up-regulated metabolites in red for females compared to males (the statistically unvarying metabolites are shown in pale grey, *p* < 0.1).

The metabolic signature of the gonads highlighted a clear sexual dimorphism, as represented in the PCA (Figure 2A1 discrimination along the *y*-axis). Similar sex-specific differences in the metabolome of mussel gonads have already been reported [47]. Volcano plots, in which individual analytes were plotted according to their respective relative fold change between males and females and their *t*-test and *p*-values, indicated that among the 2548 analytes (annotated and not annotated) observed in the gonads, 21% showed significantly different concentrations between males and females, with 245 being significantly lower and 299 significantly higher in females compared to males (Figure 2A2). PCA performed with the analytes from the digestive gland revealed slight discrimination of metabolomes of males and females along the second component axis (Figure 2B1), but weaker than the one observed in the gonads. The volcano plots showed that among the 2567 analytes detected in the digestive glands, 6% had different concentrations between the sexes, with 33 having lower concentrations and 124 higher concentrations in females than in males (Figure 2B2). No obvious sexual dimorphism was observed in the gills by PCA (Figure 2C1) and only 4.2% of the 2530 analytes presented differences in concentration between the two sexes (Figure 2C2). In the mantle, no clear sexual dimorphism was observed by PCA (Figure 2D1), but 50 of the 2301 detected analytes had lower concentrations and 115 higher concentrations in females than in males, representing a 7.1% sex-discriminated pattern (Figure 2D2). Interestingly, in three out of the four analyzed tissues, females showed significantly higher concentrations of analytes than males (299 vs. 245 in the gonad, 124 vs. 30 in the digestive gland, and 115 vs. 50 in the mantle). Overall, female organs exhibited higher analyte contents than males, suggesting that more intense and/or more complex metabolic activities may occur in females, potentially in relation to their higher reproductive effort resulting in a richer integration of reserves in female oocytes than in spermatozoids.

**Figure 3:**
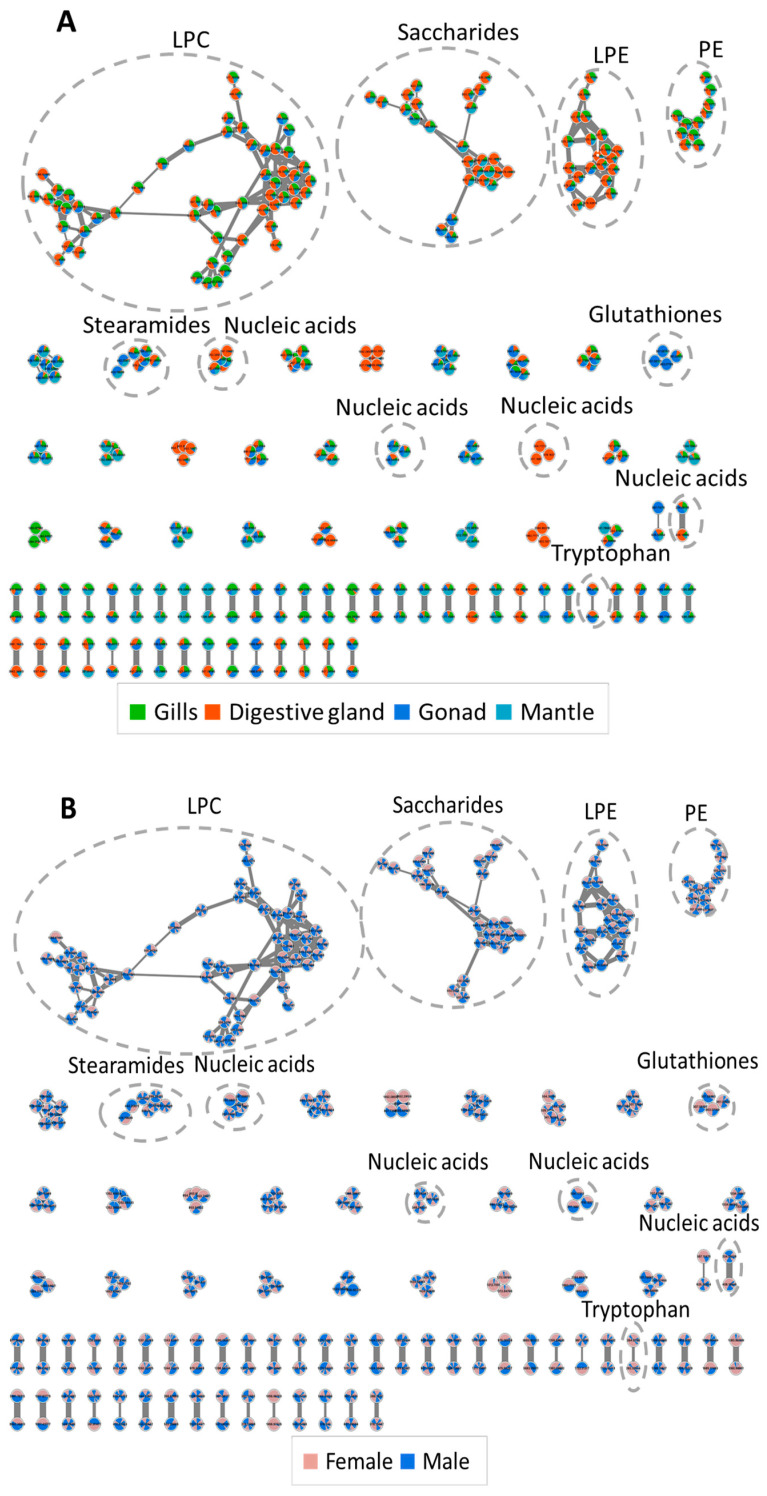
Molecular networks generated from 579 MS/MS spectra obtained from the gonads, digestive glands, gills, and mantle of *D. polymorpha* males (n = 12) and females (n = 12), using the GNPS and t-SNE tools. The diagram displays connected metabolites sharing a structural similarity based on the similarity of their respective fragmentation patterns. Annotated compounds are indicated in the dashed-line circles. Relative concentrations of annotated compounds are indicated in (**A**) each tissue (in green for gills, in red in digestive glands, in blue in gonads and in turquoise in mantles), and (**B**) in each sex (in blue in males and in red in females).

The molecular networks were identified using the GNP algorithm from MetGem software with the 579 MS/MS spectra of the most concentrated analytes detected in the gonads, digestive glands, gills, and mantle of *D. polymorpha* males and females (Figure 3). The appearance of analytes with common MS/MS fragments within the same clusters connected analytes sharing a structural similarity based on their respective fragmentation patterns (cosine score < 0.7). Among all the analytes, MetGem aligned some in parallel with annotated metabolites from public databases based on accurate mass correspondence and sharing of MS/MS fragments. Thus, based on correspondence with both the mass and the isotopic MS/MS fragmentation pattern, we were able to annotate various analytes as genuine or potential phospholipids such as lysophosphatidylcholine (LPC) principally found in the digestive glands, gills and gonads of males and females (Figure 3). Analytes annotated as lysophosphatidylethanolamine (LPE) were also abundant and located mainly in the digestive gland and the gills, and to a lesser extent in the gonads; phosphatidylethanolamine (PE) was also found at relatively high concentrations. Previous studies also reported the presence of LPE, LPC and PE in freshwater molluscs, with the major phospholipid classes being the choline-containing PC and the amine-containing PE, constituting around 50% of the total [51,52]. In *D. polymorpha*, LPE and PE constituted 47% of the total annotated phospholipids [51]. LPC is a structural lipid produced by the digestive gland, derived from phosphatidylcholine and used to build cell membranes. LPE is also a membrane component but is additionally implicated in cell signalling and enzyme activation, whereas PE is particularly abundant in the internal layer of the cellular membrane. Different saccharides were also abundant in male and female organs like the digestive gland, the gonads and the gills (Figure 3B). Saccharides are primary metabolites associated with numerous biological processes and functions in molluscs, such as growth, reserve storage, tissue architecture, immunity, energetic metabolism, and energy storage; they form a major part of mollusc tissue extracts [53]. Saccharides present a large structural variability depending on mollusc species and organ [53], as also suggested by the present study showing different molecular clusters of saccharides specific to the gonads (blue) compared to the gills (green) of *D. polymorpha* (Figure 3B). The specificity of saccharides within the gonads was especially marked in females, as seen in the molecular network diagram (Figure 3B).

Interestingly, the molecular network representation also revealed that glutathione was predominantly present in the gonads, with higher concentrations in females (Figure 3). Glutathione (GSH) is a ubiquitous antioxidant tripeptide implicated in detoxification and cellular homeostasis. Previous studies reported that gonadal tissues of bivalves contained high GSH concentrations relative to other tissues, particularly during reproductive periods. GSH helped to maintain the health of the gonads and protected the gametes from oxidative damage during fertilization and development [54,55]. A high GSH content in the gonads has been reported to ensure reproductive success in oysters, *Crassostrea virginica* by decreasing the susceptibility of gametes and embryos to metal toxicity [55]. In vertebrates, a high GSH content in the gonads during oocyte maturation is a reliable indicator of oocyte viability, as it is essential for male pronucleus formation during fertilization and embryo pre-implantation, ensuring embryo development and preventing embryonic cellular apoptosis [56]. The bivalves used in our study were at the onset of gametogenesis (January) [57], which may have influenced the GSH contents stored in the gonads for oocyte protection.

### 3.2. Analysis of Annotated Analytes from Male and Female D. polymorpha Tissues

**Figure 4:**
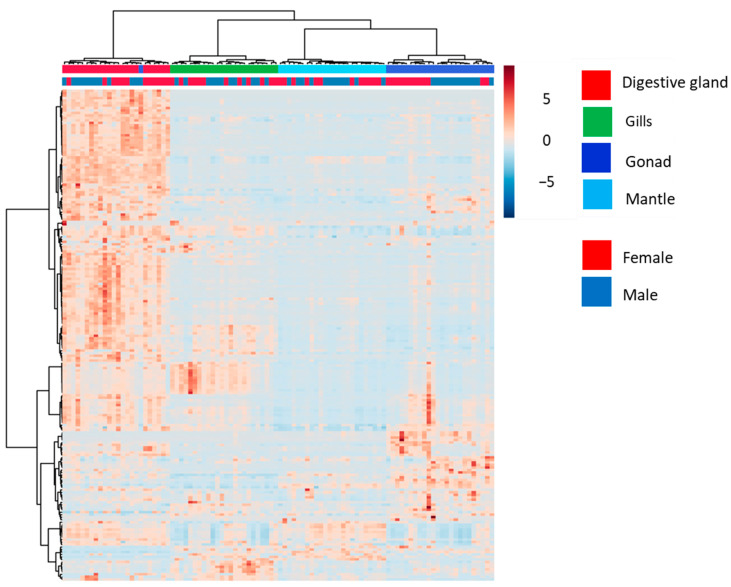
Heatmap with hierarchical classification of 198 putatively annotated metabolites measured by MS/MS in the digestive glands (red), gills (green), gonads (dark blue), and mantle (light blue) of male (n = 12) and female (n = 12) *D. polymorpha*.

The heatmap with hierarchical clustering of 198 putatively annotated analytes identified by MS/MS in the four organs of male and female mussels (Figure 4) revealed the same general discriminative patterns seen with the whole set of 2634 analytes previously observed (Figure 1). Globally, the metabolome of each tissue significantly differed from those of the other tissues (PERMANOVA *p* < 0.01, Table 2A). The heatmap also revealed that the metabolic profiles were first discriminated among the four tissue types and second between males and females (Figure 4). Considering only the 198 annotated analytes, no obvious sex-related differences were seen in the mantle and in gills, whereas a slight discrimination in the digestive gland was visually detectable on the heatmap, although it was not statistically significant (pairwise PERMANOVA *p* > 0.05, Table 2B). Only the gonads showed a clear molecular sexual dimorphism (pairwise PERMANOVA *p* < 0.05, Table 2B), but to a lesser extent than what was seen in the previous analysis (annotated and not annotated). In general, the distribution pattern of putatively annotated analytes appeared globally representative of the one observed with all analytes.

**Figure 5:**
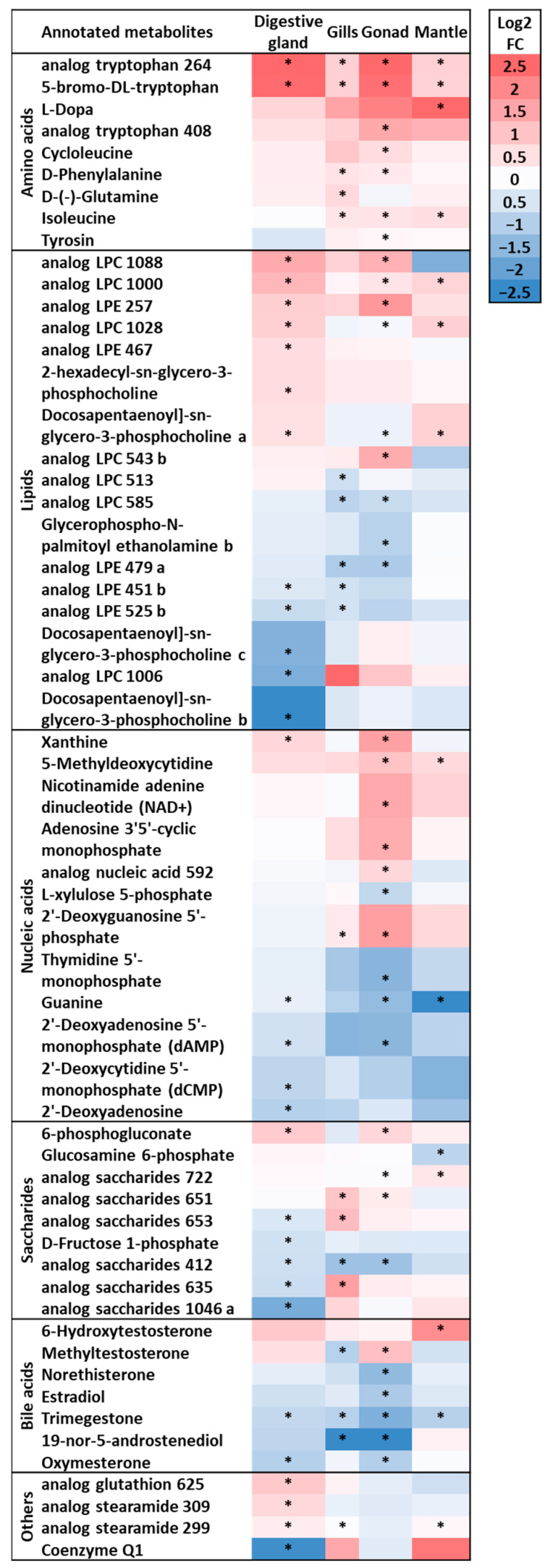
List of most discriminated metabolites among the 198 annotated molecules between males and females according to their molecular family and to the different tissues. The metabolites with concentrations higher in females are shown in red, while those higher in males are shown in blue. The darker the color the greater the fold change. Statistically significant results (ANOVA, *p* < 0.01) are indicated with a star.

Overall, the gonads presented the largest number of significantly discriminated analytes (n = 36) between males and females among the 198 putatively annotated analytes. All observed analyte classes were discriminated, especially those comprising various nucleic acids, lipids and amino acids (Figure 5). The highest number of discriminated analytes occurred in females, especially for amino acids and nucleic acids. For example, eight annotated amino acids among the nine significantly discriminated exhibited higher concentrations in females than in males, mostly in the gonads (Figure 5).

The digestive gland contained 31 significantly discriminated analytes, principally represented by lipids (equally abundant in males and females, depending on the molecule), saccharides, and nucleic acids (most abundant in males). Those analytes whose concentrations differed between the sexes were probably involved in gender-specific metabolic pathways. The gills and the mantle contained the lowest number of significantly sexually discriminated analytes (19 and 15, respectively) with higher concentrations of amino acids in females and higher concentrations of lipids in males in gills, and mostly higher concentrations of amino acids and structural lipids in female mantles. The observation that male and female mussels showed several sex-specific modulated metabolites suggests that they may have different protective mechanisms against various stresses. These sexual differences may result in substantial physiological changes, but could also influence measurable biomarkers or targeted toxicological endpoints and induce data variability, particularly if the gonads or digestive glands are investigated. Now, further studies on the metabolome of digestive glands and gonads of both sexes would be interesting to describe the metabolic differences and thus increase our understanding of sex-specific responses to specific pollutants and their ecotoxicological consequences on natural populations. The genetic background of zebra mussel populations is already known to act as a confounding factor in studies of biomarkers, because of different individual responses to contamination [17,58]. Therefore, population genetics, sex of the organism, and type of tissue studied should be carefully considered prior to an ecotoxicological or physiological investigation.

Among the sexually differentiated lipids that were annotated in the present analysis, several were structural lipids belonging to LPE and LPC classes, which account for about half of the known phospholipids in freshwater bivalves [51]. Among the top 25 most discriminated analytes regardless of tissue type, three LPCs were at higher concentrations in females, whereas two LPEs were at higher concentrations in males (Figure 5). These intrinsic biological differences in lipid content between male and female mussels may be associated with their specific needs during the reproductive cycle. This specificity would result in unique differences in their respective lipid regulation process induced by exposure to stress. A previous study performed on the marine mussel, *Mytilus galloprovincialis*, exposed to polluted effluents showed a gender-specific modulation of various LPCs and LPEs, with a general trend of up-regulation in males and down-regulation in females [59].

**Figure 6:**
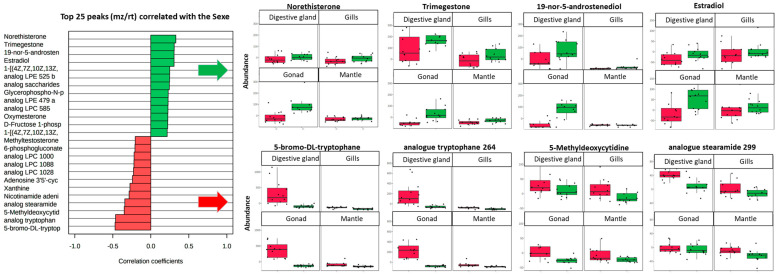
The top 25 most discriminated metabolites among the 198 annotated molecules between *D. polymorpha* males and females. The metabolites expressed principally in males are shown in green and in females in red. Abundances (mean area of the MS peak ± SD, n = 12) of the top four metabolites in the digestive glands, gills, gonads, and mantle are depicted in box plots (green for males and red for females).

Among all tissues, but mainly in the gonads, the concentrations of five cholic acid derivatives, the putatively named methyltestosterone, norethisterone, estradiol, trimegestone, and 19-nor-5-androstenediol, and oxymesterone were higher in males (Figure 6). Many cholic acid derivatives were found in the top 25 most discriminated analytes regardless of tissue type (Figure 6). The presence of molecules related to the steroid hormones that play a critical role in vertebrate reproduction has been previously observed in molluscs [60,61]. Their origin and function are still debated, but these potential sexual hormones are either thought to be absorbed through the diet or synthesized by the molluscs themselves from the steroid precursors, cholesterol or pregnenolone [62,63,64]. Molluscs have been shown to share some steroidogenic and steroid metabolic pathways with vertebrates. Three steroids, progesterone, testosterone, and 17β-estradiol, have been proposed as functional hormones in gastropods and bivalves [65,66], including *D. polymorpha* [67,68]. Such gender-specific differences in concentrations of endocrine-active metabolites may influence the response of *D. polymorpha* to contaminant exposure. However, it remains important to note that the putative annotation obtained from the present investigation cannot be considered as a genuine molecular identification, but rather provides insights into the molecular structure of these analytes as cholic-acid-related components, according to the annotations from spectral databases. Various isobaric cholic-acid-related derivatives can have the same mass and similar fragmentation patterns, and therefore cannot be differentiated in our present analysis because of the lack of specific spectral information for these molecules in public databases. Thus, the hypothesis that these gender-specific metabolites could be steroid compounds acting as potential sexual hormones or prohormones involved in steroid metabolic pathways in *D. polymorpha* remains to be confirmed.

Our study showed that several lipids could be discriminated between male and female organs in the sexual maturation (pre-spawning) period, and we predict a quantitative or qualitative evolution of these sexual differences along the entire spawning cycle of the zebra mussel. The lipid content in dressenids has already been shown to fluctuate annually in synchrony with the gametogenic cycle, exhibiting high concentrations of lipophilic organic compounds in fully mature individuals during late spring and summer, and a decrease in the post-spawning period [69].

Sex hormone concentrations in some bivalves are also dependent on gonadal stage and seasonal variation, suggesting their possible role as endogenous modulators of gametogenesis [66,70]. It would be worthwhile to characterize the sexual molecular dimorphism in zebra mussels along the entire reproductive cycle. This information might allow us to compensate for intrinsic confounding factors related to an organism’s maturity cycle when performing specific analyses on males and females using sexually dimorphic organs like gonads and digestive glands of *D. polymorpha*. Sexual molecular dimorphism in *D. polymorpha* should also be investigated in a natural setting (caged), as it is presumed that environmental factors such as temperature, food availability, and pollutants may influence the (eco)toxicological response [29,68,69].

## 4. Conclusions

A deeper knowledge of the sexual molecular dimorphism of a model animal may help strengthen ecotoxicological research through a better understanding of the observed variability of molecular responses to various environmental stressors. This study revealed a significant sexual molecular dimorphism in the gonads, and to a lesser extent in the digestive glands, of the ecotoxicological model organism *D. polymorpha* (zebra mussel). Such differences in the metabolome may influence biomarker responses to anthropogenic pollution or abiotic and biotic stresses and induce bias in interpreting data if the gonad or the digestive gland is targeted for analyses without regard to sex. Sexing individual mussels prior to sampling is difficult, but the risk of errors in data interpretation can be reduced by following physiological responses in gills or in the mantle for lowering inter-sex molecular dimorphism influences. Data provided by the present study may help in developing specific protocols and data interpretation for ecotoxicological or biosurvey investigations using *D. polymorpha*. Information on the various metabolite concentrations among organs from both sexes may also be a powerful tool for identifying new molecules of interest and increasing basic knowledge for further physiological or biomarker investigations.

## Figures and Tables

**Table 1:** Results of PERMANOVA analyses applied to the MS/MS peak list of 2634 analytes for comparison of their relative quantity according to tissues (A) and sexes (B). Significant *p*-values are indicated in bold (*p* < 0.05).

(**A**)			
**Tissues**	**PERMANOVA**		
	**F.Model**	**R^2^**	***p*-Value**
	28.634	0.48286	**0.001**
**Pairwise Tissues**	**Pairwise PERMANOVA**		
	**F.Model**	**R^2^**	***p*-Value**
**Digestive gland vs. mantle**	60.33	0.57	**0.001**
**Gills vs. digestive gland**	28.22	0.38	**0.001**
**Gills vs. mantle**	26.99	0.37	**0.001**
**Digestive gland vs. gonads**	26.81	0.37	**0.001**
**Gonads vs. mantle**	19.85	0.3	**0.001**
**Gills vs. gonads**	14.11	0.23	**0.001**
(**B**)			
**Sex**	**PERMANOVA**		
	**F.Model**	**R^2^**	***p*-Value**
	13.989	0.52669	**0.001**
**Pairwise Female vs. Male**	**Pairwise.PERMANOVA**		
	**F.Model**	**R^2^**	***p*-Value**
**Gonads**	3.59	0.14	**0.001**
**Digestive gland**	1.61	0.07	**0.042**
**Mantle**	1.14	0.05	0.272
**Gills**	1.03	0.04	0.317

**Table 2:** Results of PERMANOVA analyses of the MS/MS peak list of the 198 putatively annotated analytes for comparisons of their relative quantity according to tissue (A) and sex (B). Significant *p*-values are indicated in bold (*p* < 0.05).

(**A**)			
**Tissues**	**PERMANOVA**		
	**F.Model**	**R^2^**	***p*-Value**
	33.193	0.51978	**0.001**
**Pairwise Tissues**	**Pairwise.PERMANOVA**		
	**F.Model**	**R^2^**	***p*-Value**
**Digestive gland vs. mantle**	71.45	0.61	**0.001**
**Gills vs. mantle**	38.68	0.46	**0.001**
**Digestive gland vs. gonads**	32.07	0.41	**0.001**
**Gills vs. digestive gland**	28.14	0.38	**0.001**
**Gonads vs. mantle**	18.14	0.28	**0.001**
**Gills vs. gonads**	17.47	0.28	**0.001**
(**B**)			
**Sex**	**PERMANOVA**		
	**F.Model**	**R^2^**	***p*-Value**
	15.433	0.55109	**0.001**
**Pairwise Female vs. Male**	**Pairwise.PERMANOVA**		
	**F.Model**	**R^2^**	***p*-Value**
**Gonads**	2.65	0.11	**0.048**
**Digestive gland**	1.38	0.06	0.207
**Gills**	0.63	0.03	0.587
**Mantle**	0.47	0.02	0.930

## Data Availability

The datasets generated and analyzed during the current study are available in the Mendeley data repository (https://data.mendeley.com/datasets/ghhxtb45mm/1 (accessed on 20 September 2022)).
